# Face with Mask Detection in Thermal Images Using Deep Neural Networks

**DOI:** 10.3390/s21196387

**Published:** 2021-09-24

**Authors:** Natalia Głowacka, Jacek Rumiński

**Affiliations:** Department of Biomedical Engineering, Faculty of Electronics, Telecommunications and Informatics, Gdansk University of Technology, Narutowicza 11/12, 80-233 Gdansk, Poland; jacek.ruminski@pg.edu.pl

**Keywords:** thermal images, face detection, face with mask detection, deep neural networks

## Abstract

As the interest in facial detection grows, especially during a pandemic, solutions are sought that will be effective and bring more benefits. This is the case with the use of thermal imaging, which is resistant to environmental factors and makes it possible, for example, to determine the temperature based on the detected face, which brings new perspectives and opportunities to use such an approach for health control purposes. The goal of this work is to analyze the effectiveness of deep-learning-based face detection algorithms applied to thermal images, especially for faces covered by virus protective face masks. As part of this work, a set of thermal images was prepared containing over 7900 images of faces with and without masks. Selected raw data preprocessing methods were also investigated to analyze their influence on the face detection results. It was shown that the use of transfer learning based on features learned from visible light images results in mAP greater than 82% for half of the investigated models. The best model turned out to be the one based on Yolov3 model (mean average precision—mAP, was at least 99.3%, while the precision was at least 66.1%). Inference time of the models selected for evaluation on a small and cheap platform allows them to be used for many applications, especially in apps that promote public health.

## 1. Introduction

Face detection in thermal images using deep neural networks is still a challenge because of small amount of thermal images needed for algorithms learning. The biggest advantages of using the thermal domain is possibility of detecting during night and day and independence from weather and illumination condition. The advantages of using thermal images are used, among others, for face detection and tracking [1], face recognition [2], analysis of local temperature changes [3,4] or estimation of biomedical signals [5,6]. Low resolution thermal cameras become more available and the features obtained from such thermal images can be improved using deep-learning-based algorithms such as super-resolution algorithms [7] or transformers [8].The use of thermal imaging in conjunction with deep learning, using available and cheap thermographic cameras and small modules containing GPU, will allow quickly analyzing images and create low-cost systems, for example for remote health control.

### 1.1. Literature Review

There are some algorithms developed primarily for face detection in thermal images—Projection Profile Analysis (PPA) [9] or Eye Corner Detection (ED) [10]. These algorithms are well suited for detecting in images where the person is dominant object in the image and its whole head and upper part of torso are visible.

In computer vision tasks, face detection is one of basic, the most popular and well known issue. In visible light images spectrum are a lot of solutions for this task, including many of them are based on deep neural networks. Of course, there are also traditional solutions, including Viola-Jones [11], but now classical methods are often replaced by newer solutions based on deep learning. Zhang et al. [12] designed face detector named FaceBoxes. This convolutional neural network model was created to have superior performance on both speed and accuracy. The speed of FaceBoxes is unchanging to the number of faces in the picture. It achieves a good accuracy in comparing with another face detection methods. Face detection can also be performed on the basis of well-known models designed for object detection. Yang and Jiachun [13] applied YOLO model to face detection. The model was trained on WIDER FACE data set [14]. Using YOLO the speed of detection can meet real-time detection requirements. From the other side, face detection can be performed in conjunction with a face recognition task. This multi-task solution was used in F-DR Net [15]. F-DR Net has a parallel architecture and detection network based on FaceBoxes model. This method was evaluated on several face detection and recognition datasets and it has better recognition and detection accuracy than other methods. Several deep-learning-based approaches focused on face and face mask detection have been investigated in [16]. The study was based on visible light images demonstrating high classification accuracy using the fine-tuned VGG-16 model.

For face detection in thermal images, such as in the visible spectrum, algorithms or machine learning-based approaches can be used. In [17], authors compare five algorithms which have been successfully applied for face detection in visible light images with algorithms especially designed for detection in thermal images. The Haar cascade classifier [11], Histograms of Oriented Gradients (HOG) [18] and the other can be used for detection in thermal images if trained with a well-fitting database. On the other hand, when deep neural networks are more efficient, it is naturally to use this solution for face detection. Attempts to adapt the neural networks used with visible light images have brought many successes. One of the models commonly used for face detection in thermal imaging is Yolov3 [19]. This model was made for object detection and its most salient feature is making detection at three different scales—small objects can be well detected because of preserving the fine grained features. Yolov3 was adopted for face detection in thermal images for in-vehicle monitoring of passengers in [20]. The results of authors experiments on their test set show an AP_50_ of 99.7% and an AP of 78.5%. In [1], the authors analyze using deep learning algorithms for face detection in low resolution thermal sequences. The Inception v3 model [21] was used—it is a great model for object classification, but it was trained on visible light images. To resolve this problem, transfer learning technique can be used for re-training the Inception v3. The model returns only class probabilities, so its last layer should be modified for object detection task. The changes introduced to the model allowed it to be successfully adapted to detection on thermal images. Thermographic images are also used for tasks other than face detection. Peng et al. in [22] created NIRFaceNet—a convolutional neural network modified from GoogLeNet [23]. It has fewer parameters than previously designed AlexNet [24], but model achieving higher accuracy. The experimental results compared to other solutions—such as Local Binary Pattern + Principal Component Analysis (LBP + PCA) [25] or GoogLeNet, demonstrate that the proposed architecture has an overall advantage, especially when image has blur or noise. In the other hand, thermal images are used for face authentication [26]. The proposed neural network architecture consists of 12 sequential layers, which takes as input one channel image. The data set used includes faces in different positions, lightning conditions and emotions, that gives the model the ability to recognize in any condition. In compared to state-of-the-art thermal face recognition algorithms (like NIRFaceNet or GoogLeNet), the proposed method achieves better recognition accuracy (99.6%). Another algorithm created for thermal face recognition tasks was made by Wu et al. [2]. Proposed model optimizes the neural network structure through the local receptive field, power sharing and sampling. Using the RGB-D-T face database [27] for CNN testing and comparing with other methods such as LBP, HOG in categories: head rotation, expression, and illumination are conditions. In all categories, the method created by us has the best thermal recognition accuracy.

### 1.2. Contribution

In this paper, we explored whether and how the face detection algorithms designed for detecting in visible light can be adapted for thermal images of a face with and without a virus protective mask work. We analyzed four face detection algorithms with different base models using about 8000 thermal images that we collected with four different thermal cameras. Additionally, we analyzed the role of different thermal raw data preprocessing methods on face detection quality metrics. We check whether using a cheap and small module equipped with a GPU, it is possible to achieve a satisfactory inference time for use in everyday applications (e.g., for remote temperature control). Finally, we investigated the role of transfer learning using features learned on visible light images.

## 2. Materials and Methods

Due to the lack of access to a large number of thermal images of the face especially with virus protective masks, we decided to create our own image database. The database contains thermal images of the face and their descriptions (location in the image and information on whether the person wear face mask, face visor or be without them). The experiment was performed with permission of local Committee for Ethics of Research with Human Participants of 02.03.2021.

### 2.1. Description of Cameras

Thermal sequences were collected using several cameras to obtain more varied dataset. The descriptions of used cameras are shown below in Table 1.

### 2.2. Image Acquisition Methods

**Figure 1:**
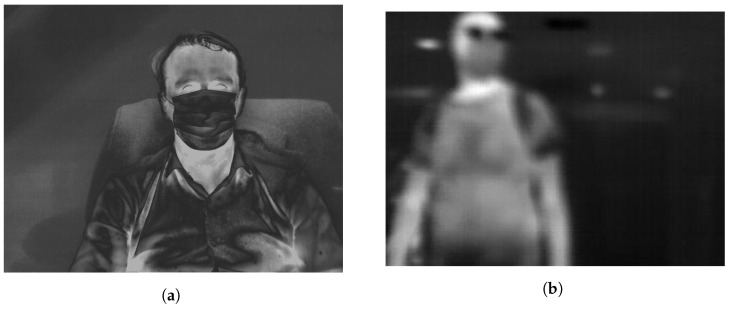
Examples of two types of images included in dataset: (**a**) good quality image and (**b**) poor quality image.

Using various types of cameras, we recorded thermal sequences from which individual images was extracted. These sequences depicts people who’s entering the building (these people should wear different types of masks, but also can be without them), people moving towards the camera, and the faces of people with and without a mask making head movements (a side-to-side and an up-and-down movements) at two different distances from the cameras (60 cm and 250 cm). Due to different types of recordings, the faces shown in the pictures are in different positions and in different scales. Recorded thermal sequences contain both good and poor quality images. Examples of such images are shown in Figure 1.

### 2.3. Dataset

The dataset consisted of 7920 images. It contains 7285 images where people are wearing a mask and 635 images where people are without one. The created dataset is not balanced, because we were interested in images with masks and images without masks only for reference. The entire dataset includes 10,555 face labels, since some images had more than one face. Among the collected images, women accounted for 42% of participants in the experiment, and men for 58%. The average age of people was 26.42 years. The created dataset was divided into training (90%) and test sets (10%). The test set includes images captured by each camera and images with and without masks.

From test set we extracted two smaller sets—one containing images with mask and second containing images without. The first one include 6% of collected images and the second one—4%. After that, we obtained three test sets—original, with mask and without mask.

### 2.4. Annotations of Images

After collecting thermal sequences, we extract some frames of every sequences. For FLIR A655SC and SC3000 frame offset was set to 20 (which means that every 8th frame was annotated), for FLIR Boson to 10 and for FLIR A320G to 8. To describe them, we have created a web application that allows to create annotations, which was based on websocket technology. For showing frames from a sequence, every frame was converted using normalization to 0–255 range, next saved as grayscale image and shown during annotation. For each frame, the description includes the location of each faces in the image and information whether a face mask (or face visor) is or not on the face. The criteria for annotating the face were: marking the regions with rectangles that include the forehead, the chin and the cheeks, and a region could be marked if a minimum of 50% of its area and two eyes were visible. When describing the images, if the annotator was unsure that the face mask was on, it should mark that the person has not it on the face. Additionally, to mark the region with ‘face mask’ label, the face mask should cover at least mouth. Annotation of images was made by eight people. Face is relatively easy to recognize by the experts, so we assume that annotations of faces were done correctly.

### 2.5. Data Preprocessing

**Figure 2:**
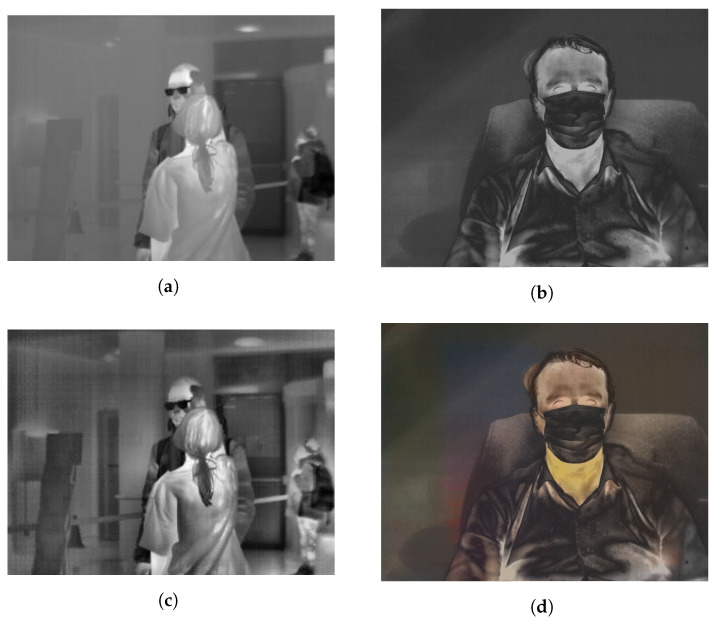
Example of three types of preprocessing: (**a**,**b**) images with normalized range of raw data, (**c**) image is image (**a**) after changing contrast using CLAHE and (**d**) image is image (**b**) after colorization.

Recorded sequences were saved as raw data. This type of saving sequences allows for extensive preprocessing of data that will be used for model training and testing. The first step is to normalize the entire range of raw data values to 0–255 for each frame. The second version of data preprocessing is to change the contrast of images using Contrast Limited Adaptive Histogram Equalization with 8x8 window. This type of histogram equalization is added to images with original values range of raw data, which are next normalize to 0–255 range (like in first type). The last one type of image preprocessing is to use image colorization to the images in dataset. All of analyzed algorithms were designed to work with images registered in visible light. Image colorization of thermal images should increase accuracy achieved by the models. It was done with the use of a solution proposed by the authors of the article [28]. An example of three data preprocessing cases are shown in Figure 2.

Due to the use of three different data preprocessing methods, the original test set was also presented in three versions: original (normalized), with CLAHE and with colorization.

### 2.6. Adaptation of Deep Learning Models

We have decided to analyze four deep learning face detection models with different base models, which originally was designed to detect faces in visible light images. This will allow us to analyze the effectiveness of deep-learning-based face detection algorithms applied to thermal images, especially for faces covered by virus protective face masks.

First of chosen models is UltraLight model [29]. This model was designed for face detection and for being lightweight for using in edge computing devices. The authors provided two versions of model: version-slim (with network backbone simplification, slightly faster) and version-RBF (with the modified RFB module and higher precision). The size of the model is approximately 1MB. For these two versions, the hyperparameters were the same and are listed in Table 2. For this model, learning rate was reduced by 0.1 after 95 and 150 epoch.

RetinaFace [30] is the second model selected for testing. This model was originally created using Mxnet library, but we use its implementation in PyTorch [31]. The authors provided two versions of backbone net—MobileNet-0.25 and ResNet-50. Base models hyperparameters are shown in Table 2 below. The models was trained using SGD as optimization method with 0.9 momentum, weight decay at 0.0005. Initial learning rate was divided by 10 at 190 and 220 epoch in Mobilenet-0.25 version and at 150 and 190 epoch in ResNet-50 version.

The next one was the Yolov3 model [19]. Before starting the selection of the type of Yolo model, preliminary tests for thermal images were carried out between versions: 3, 4, and 5. As the best results were obtained for the Yolo model in version 3, it was selected for analysis. Its implementation in PyTorch was used. The optimization method is SGD with 0.937 Nesterov momentum and 0.0005 weight decay. The remaining model hyperparameters are included in Table 2. At the beginning of the training process, a warm-up (3 epochs) is performed. The learning rate is changed with cosine function.

LFFD: A Light and Fast Face Detector for Edge Devices [32] was the last selected for testing. This detector balancing both accuracy and running efficiency. The testing task will use the second version (v2) of the model (not presented in the article). In this version, detection scale is 10–320 and the backbone is modified for faster inference compared to the version presented in the paper. SGD with 0.9 momentum was used as optimization method and 0.00001 weight decay. The model was trained over 1,000,000 iterations, and the learning rate was reduced by dividing by 10 the current value at the 300,000, 600,000 and 900,000th iterations.

Each model additionally introduces its own image preprocessing. The Yolov3 model uses a random change of perspective and random changes in color, hue, saturation, and brightness. The LFFD model also uses random changes in saturation and brightness, but also in contrast, random horizontal and vertical flip, and blur. Classic preprocessing, like brightness, contrast, saturation, and hue random changes, is used by RetinaFace too. In addition, image cropping, mirroring or resizing to a given dimension are also used. The last model—the Ultra Light model—uses the same methods as in the RetinaFace model, but also randomly adds noise to the image.

### 2.7. Models Testing Scenario

**Figure 3:**
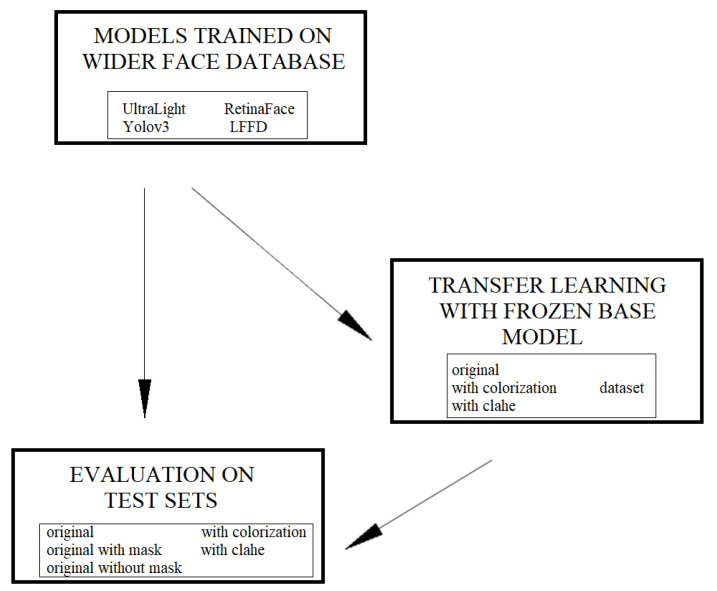
The framework model of the testing scenario.

We decided to carry out two different approaches to testing selected models. Figure 3 shows the framework model of the testing scenario. The first approach was to see how each model performs face detection in thermal images when it was trained on the visible images dataset. In our case it was the WIDER FACE dataset for all models. The second approach involves the use of already acquired knowledge while training models on a set of visible images—transfer learning. In this case, we use a model that has already been trained and we fine-tuned it using the database created by us. The use of transfer learning consists of taking a model previously trained on a set of visible images (in our case on WIDER face database), then we use freezing the layers of the basic model and train the last layers to adapt model to work with thermal images.

For each of the approaches, each model will be evaluated on the three created test sets.

The last element of the evaluation of selected models will be to measure the inference time to check if it is satisfactory enough to use the models in applications in everyday life. The inference time will be measured for images with two resolutions—640 × 480 and 320 × 240 pixels, using the NVIDIA Jetson Nano module (with NVIDIA Maxwell architecture) and DGX-1 station (on one NVIDIA Tesla V100 SXM2 32GB GPU).

### 2.8. Metrics

To measure the performance of the analyzed models we have choose three metrics: mean Average Precision (mAP) [33], precision, and recall [34]. They are a popular metrics for measuring accuracy of object detectors. Precision measures accuracy of predictions (percentage of correct predictions), while recall measures the number of correct positive prediction in all positive prediction. Average precision combines precision and recall (calculating the area under the curve (AUC) of the Precision x Recall curve) and the higher the score is, the higher performance the model has.

## 3. Results

Table 3, Table 4, Table 5 and Table 6 show the results obtained for each test scenario. The results obtained for each model and test set are the average of three test approaches in order to avoid randomness of the obtained results. The use of models learned only on images recorded in visible light does not give particularly high and desirable results in the case of thermal images. Compared to other models, the Yolov3 model is distinguished by a particularly high value of the mean average precision (mAP), precision, and recall. Moreover, for the remaining models, the collected metrics for the original test set with faces only in masks were significantly lower than for the mixed sets, and for the set with images of faces without masks, they reached the highest values.

The use of transfer learning has shown satisfactory results for the UltraLight, LFFD, and RetinaFace models. Using the original training set, the best results are achieved by the Ultra Light model, comparable for the slim and RBF versions. When the contrast enhanced image set using CLAHE was selected as the training set, in addition to the Yolov3 model, the Ultra Light model performs well with face recognition, especially for the RBF version, where the mAP is over 80%. The use of the training set with CLAHE, compared to the basic, original training set, resulted in an improvement of the mAP metric for the RetinaFace model—using the MobileNet model and for the Ultra Light model in the RBF version.

The use of colorization of images to resemble images recorded in visible light has not worked for every of analyzed model. The improved metrics are noticeable for the Retina Face model and for the Ultra Light model in the RBF version (compared to the original training set). The Yolov3 model shows a similar mean average precision, regardless of the training set used, but the precision of this model is lower when used images after colorization.

**Figure 4:**
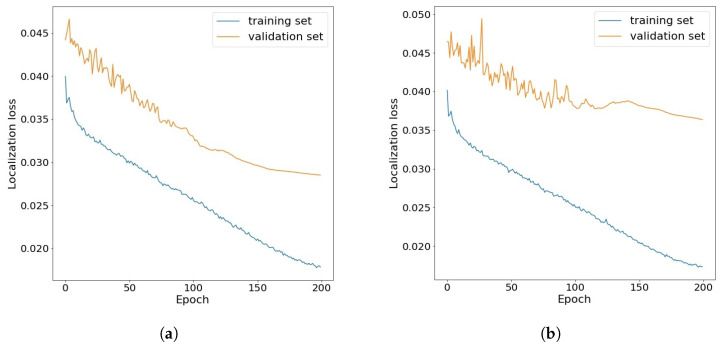
Example of localization loss change during transfer learning for Yolov3 model: (**a**) on the own original dataset (**b**) on the CLAHE dataset.

Figure 4 shows the losses obtained for the training and validation sets during the transfer training of the Yolov3 model using the original set and the CLAHE data set. The loss function shown in the charts is the localization loss, which illustrates the error between the predicted boundary box and the ground truth. On the basis of these two examples, it can be seen that for the training set, both in the original set and in the CLAHE set, it decreases with successive epochs. For the validation subset of these two datasets, the loss is higher, but over the course of almost the entire learning cycle, it decreases with subsequent epochs.

Table 7 and Table 8 present the results of the measured inference time for images with two resolutions—640 × 480 and 320 × 240 pixels. These are two of the resolutions of the images that were collected in the created dataset. When analyzing the obtained results, it can be seen that for the Jetson Nano module, the UltraLight, RetinaFace and LFFD models achieve satisfactory inference times. The use of a relatively cheap and small module can be used when creating applications that will be used in everyday life. Compared to the measured inference time on the DGX-1 computer, the times achieved by the Jetson Nano module are very satisfactory and confirm the possibility of using this solution without large wastage of time (both for low and high resolution). The Yolov3 model, despite achieving the best evaluation metrics, does not have the best inference time. In practical applications, models with a lower inference time will be more desirable—this will allow for faster image analysis and obtaining the result.

## 4. Discussion

The use of transfer learning had a positive impact on the obtained results. For all models, they improved compared to those achieved for models trained only on the set of visible images. When analyzing the impact of preprocessing on the results obtained, the use of CLAHE also had a positive effect on the Ultra Light model in the RBF version and on the RetinaFace model with MobileNet backbone. Applying colorization to thermal images was to help check whether, for models designed to work with images made in visible light, it would help to increase the measures achieved by the models. However, it did not bring much improvement, perhaps due to the fact that the colors were not perfectly matched, many of the images were not of high quality and then the colorization was not accurate (blurred boundaries did not allow separating the entity well).

To improve the results, it would be worth considering collecting more data and training the model from scratch. In such a case, the models could achieve better parameters. Collecting only good quality data would certainly also improve the results, while in the created dataset there was both good and poor quality images (people were recorded in motion; the images had blurred edges).The prepared data set includes images of different resolutions. Certainly, the use of only high resolution images would improve the results achieved by the models.

The analyzed models were designed to operate on images recorded in visible light. In our case, we test thermal images, mostly with masked faces (92% of the images in the dataset had faces covered with masks). Using transfer learning on models that have been learned in images with uncovered faces, some of the information that is used by them (e.g., nose, mouth—characteristic features of a face) is not visible in the images from our set.

Another approach would be to create a model dedicated to face recognition in thermal images, for example based on one of the models that works well for detecting them in visible images. Adjusting and fine-tuning such a solution would certainly result in better measures achieved by the model.

The use of the dataset that was created to test this approach and creating or fine-tuning of any existing models will be applied if we use such a model in situations where people will be wearing masks (e.g., checking at the entrance to the building or in places where wearing masks is mandatory). If the model were to be more universal, more thermal images with marked faces should be collected, where people’s faces are not hidden. Additionally, face detection in thermal images can be used to determine body temperature due to the benefits of thermography. This approach will be especially useful when monitoring entrances to buildings and will allow for early detection of people with increased body temperature. With this application or in crowded places, cheap and small platforms, that allow for quick image analysis combined with good precision and sensitivity achieved by the model, can be successfully used.

In [20], the authors demonstrated the possibility of using the Yolov3 model for face detection in thermal images, but without the faces with masks. They achieved an average precision on face detection of 78.5%. They also used transfer learning to adapt the Yolov3 model to work with thermographic images. In our case, the same model was also tested for use with this type of images and in our case the mAP value was higher, up to 99.3%, but we used a larger set of images. A version of the Yolov3 model with added the SE attention mechanism module and SPP module to the Yolov3 was used in [35]. The solution tested by us for the same model also achieves higher mAP values by 17.4% and 22.8% compared to the solutions proposed by the authors, even though in our case most of the faces in the images are partially covered by the virus protective mask.

In [20], authors used 5361 labelled thermal face images captured using one type of a camera. Similarly in [36] 4200 images were used to creation a database, using only one type of a camera. In our work, 7920 thermal images of a face were collected with 10,555 face labels. It is a significant contribution since it is one of the first databases that contains thermal images with faces covered by protective masks collected using four types of thermal cameras.

## 5. Conclusions

In this work, we addressed the problem of face (with protective masks) detection in thermal images. A similar problem for visible light images was well researched. The use of thermal images is very convenient for skin temperature evaluation with better privacy preservation of subjects than for visible light images (especially when defocused/smoothed images are collected). To analyze the problem, we originally collected a set of approximately 8000 images using four different thermal cameras. Each image was annotated manually and used in face detection studies. To our knowledge it is the largest set of data with the above-mentioned characteristics. We adapted and analyzed a set of face detection algorithms that proved to be efficient for visible light images. Using standard metrics, we demonstrated that the Yolov3-based model showed the best results with mAP at least 99.3%. We also presented that almost all the analyzed face detection deep models trained only on visible light images are not suitable for face with mask detection from thermal images. We showed that most of the analyzed algorithms and related data preprocessing methods require additional training using thermal datasets. Without such training, the mAP was typically less than 30%. Transfer learning significantly improved the obtained mAP values. For example, for the very time efficient UltraLight model (e.g., 35 ms per frame) the mAP improved from about 20% to about 80%. We also showed that special data preprocessing like CLAHE, false colorization, masking, etc. is not crucial to improve the precision of face detection from thermal images.

It is possible to use thermal images for face detection in conjunction with models created and trained on images recorded in visible light. The results obtained for models that have been trained only in the WIDER FACE set are much lower than in the case of using transfer learning of these models with the data collected in our own data set. Data preprocessing, such as CLAHE or image colorization, also has positive effects, depending on the model used. A model that is good at working with thermal images, even if trained only on the set of visible images, is the YOLOv3 model, which can be successfully used to work with such images.

Future work should focus on improving the algorithms to improve the precision while keeping at least the same values of mAP/recall. It will be important to extend the dataset with new images, as well as attempt to train the model from scratch, also using various types of preprocessing. Another goal should be to create a dedicated model to work with thermal images and face detection with and without masks.

## Figures and Tables

**Table 1:** Descriptions of cameras.

Model	Manufacturer	Spatial Resolution	Dynamic Range	Frame Rate
A320G	FLIR Systems	320 × 240	16 bit	60 fps
A655SC	FLIR Systems	640 × 480	16 bit	50 fps
SC3000	FLIR Systems	320 × 240	14 bit	50 fps
Boson	FLIR Systems	640 × 512	14 bit	8 fps

**Table 2:** Models hyperparameters.

Model Name	Base Model	Number of Epochs	Batch Size	Optimizer	Initial Learning Rate
UltraLight	version-slim	200	24	SGD	0.01
	version-RBF				
RetinaFace	MobileNet-0.25	250	32	SGD	0.001
	ResnNet-50	200	24		
Yolov3	-	200	16	SGD	0.01
LFFD	-		32	SGD	0.1

**Table 3:** Results obtained for testing model trained on WIDER FACE dataset.

Trained on WIDER FACE					
Model		Dataset	mAP	Precision	Recall
UltraLight	version-slim	original	0.165	0.514	0.144
		with CLAHE	0.207	0.514	0.196
		with colorization	0.216	0.530	0.209
		original with mask	0.107	0.436	0.105
		original without mask	0.267	0.598	0.210
	version-RBF	original	0.166	0.514	0.180
		with CLAHE	0.200	0.535	0.192
		with colorization	0.222	0.539	0.260
		original with mask	0.096	0.398	0.121
		original without mask	0.286	0.640	0.281
RetinaFace	MobileNet-0.25	original	0.315	0.565	0.285
		with CLAHE	0.337	0.594	0.297
		with colorization	0.296	0.475	0.325
		original with mask	0.218	0.487	0.209
		original without mask	0.467	0.648	0.416
	ResNet-50	original	0.233	0.464	0.245
		with CLAHE	0.274	0.528	0.261
		with colorization	0.231	0.434	0.254
		original with mask	0.125	0.353	0.172
		original without mask	0.392	0.597	0.373
Yolov3		original	0.994	0.638	0.997
		with CLAHE	0.996	0.634	0.997
		with colorization	0.996	0.621	0.997
		original with mask	0.994	0.625	0.998
		original without mask	0.994	0.663	0.996
LFFD		original	0.163	0.461	0.168
		with CLAHE	0.220	0.562	0.206
		with colorization	0.172	0.439	0.193
		original with mask	0.090	0.360	0.119
		original without mask	0.287	0.570	0.252

**Table 4:** Results obtained for testing model trained on own original dataset.

Transfer Learning—Original Training Set					
Model		Dataset	mAP	Precision	Recall
UltraLight	version-slim	original	0.839	0.802	0.829
		with CLAHE	0.795	0.788	0.749
		with colorization	0.828	0.799	0.829
		original with mask	0.836	0.793	0.834
		original without mask	0.844	0.820	0.822
	version-RBF	original	0.829	0.826	0.819
		with CLAHE	0.764	0.818	0.713
		with colorization	0.836	0.835	0.818
		original with mask	0.831	0.810	0.826
		original without mask	0.827	0.855	0.807
RetinaFace	MobileNet-0.25	original	0.473	0.674	0.444
		with CLAHE	0.347	0.543	0.353
		with colorization	0.399	0.655	0.375
		original with mask	0.333	0.577	0.352
		original without mask	0.662	0.798	0.609
	ResNet-50	original	0.516	0.755	0.515
		with CLAHE	0.435	0.655	0.478
		with colorization	0.474	0.721	0.496
		original with mask	0.437	0.703	0.433
		original without mask	0.618	0.823	0.654
Yolov3		original	0.996	0.716	0.997
		with CLAHE	0.994	0.727	0.997
		with colorization	0.996	0.718	0.997
		original with mask	0.996	0.691	0.998
		original without mask	0.993	0.764	0.996
LFFD		original	0.688	0.748	0.667
		with CLAHE	0.608	0.743	0.555
		with colorization	0.685	0.753	0.659
		original with mask	0.722	0.749	0.715
		original without mask	0.645	0.743	0.585

**Table 5:** Results obtained for testing model trained on own dataset with CLAHE.

Transfer Learning—Training Set with CLAHE					
Model		Dataset	mAP	Precision	Recall
UltraLight	version-slim	original	0.780	0.805	0.778
		with CLAHE	0.780	0.802	0.794
		with colorization	0.771	0.803	0.775
		original with mask	0.795	0.805	0.792
		original without mask	0.759	0.803	0.758
	version-RBF	original	0.843	0.852	0.770
		with CLAHE	0.843	0.839	0.786
		with colorization	0.823	0.847	0.741
		original with mask	0.840	0.840	0.771
		original without mask	0.850	0.872	0.776
RetinaFace	MobileNet-0.25	original	0.506	0.704	0.472
		with CLAHE	0.507	0.700	0.476
		with colorization	0.494	0.716	0.437
		original with mask	0.386	0.622	0.385
		original without mask	0.675	0.805	0.623
	ResNet-50	original	0.469	0.729	0.494
		with CLAHE	0.565	0.767	0.562
		with colorization	0.438	0.745	0.450
		original with mask	0.385	0.666	0.416
		original without mask	0.605	0.815	0.629
Yolov3		original	0.994	0.727	0.997
		with CLAHE	0.995	0.725	0.997
		with colorization	0.994	0.731	0.997
		original with mask	0.994	0.711	0.998
		original without mask	0.994	0.761	0.996
LFFD		original	0.511	0.729	0.497
		with CLAHE	0.671	0.743	0.682
		with colorization	0.507	0.726	0.499
		original with mask	0.492	0.703	0.496
		original without mask	0.556	0.779	0.499

**Table 6:** Results obtained for testing model trained on own dataset with colorization.

Transfer Learning—Training Set with Colorization					
Model		Dataset	mAP	Precision	Recall
UltraLight	version-slim	original	0.813	0.822	0.796
		with CLAHE	0.758	0.844	0.684
		with colorization	0.800	0.821	0.787
		original with mask	0.817	0.810	0.801
		original without mask	0.810	0.843	0.790
	version-RBF	original	0.833	0.815	0.795
		with CLAHE	0.779	0.825	0.702
		with colorization	0.828	0.818	0.787
		original with mask	0.831	0.800	0.800
		original without mask	0.834	0.840	0.790
RetinaFace	MobileNet-0.25	original	0.484	0.695	0.454
		with CLAHE	0.375	0.534	0.393
		with colorization	0.468	0.702	0.438
		original with mask	0.335	0.592	0.345
		original without mask	0.687	0.807	0.644
	ResNet-50	original	0.532	0.707	0.541
		with CLAHE	0.403	0.587	0.459
		with colorization	0.537	0.724	0.560
		original with mask	0.406	0.627	0.458
		original without mask	0.691	0.825	0.682
Yolov3		original	0.995	0.643	0.997
		with CLAHE	0.993	0.639	0.997
		with colorization	0.995	0.642	0.997
		original with mask	0.994	0.616	0.998
		original without mask	0.995	0.699	0.996
LFFD		original	0.568	0.729	0.546
		with CLAHE	0.568	0.729	0.546
		with colorization	0.665	0.733	0.667
		original with mask	0.677	0.749	0.690
		original without mask	0.682	0.794	0.589

**Table 7:** Inference time for images with a resolution of 640 × 480.

Inference Time in Milliseconds—640 × 480 Images			
Model		Jetson Nano	DGX-1 Station
UltraLight	version-slim	28.57	5.80
	version-RBF	37.42	6.99
RetinaFace	MobileNet-0.25	37.16	29.74
	ResNet-50	58.52	20.07
Yolov3		685.11	3.82
LFFD		69.04	20.18

**Table 8:** Inference time for images with a resolution of 320 × 240.

Inference Time in Milliseconds—320 × 240 Images			
Model		Jetson Nano	DGX-1 Station
UltraLight	version-slim	34.44	7.34
	version-RBF	35.50	9.10
RetinaFace	MobileNet-0.25	35.63	9.04
	ResNet-50	57.95	15.26
Yolov3		325.47	4.85
LFFD		63.23	16.83
